# Comparison of Perioperative Outcomes for Prostate Artery Embolization Versus Transurethral Resection of the Prostate and Laser Enucleation for Benign Prostatic Hyperplasia: Results from the GRAND Study

**DOI:** 10.3390/jcm14176135

**Published:** 2025-08-29

**Authors:** Nikolaos Pyrgidis, Daniel Puhr-Westerheide, Gerald Bastian Schulz, Matthias Philipp Fabritius, Philipp M. Kazmierczak, Max Seidensticker, Jens Ricke, Christian Stief, Philipp Weinhold, Julian Marcon, Patrick Keller

**Affiliations:** 1 Department of Urology, University Hospital, LMU Munich, 81377 Munich, Germany; gerald.schulz@med.uni-muenchen.de (G.B.S.); christian.stief@med.uni-muenchen.de (C.S.); philipp.weinhold@med.uni-muenchen.de (P.W.); julian.marcon@med.uni-muenchen.de (J.M.); patrick.keller@med.uni-muenchen.de (P.K.); 2 Department of Radiology, University Hospital, LMU Munich, 81377 Munich, Germany; daniel.puhr-westerheide@med.uni-muenchen.de (D.P.-W.); philipp.kazmierczak@med.uni-muenchen.de (P.M.K.); max.seidensticker@med.uni-muenchen.de (M.S.); jens.ricke@med.uni-muenchen.de (J.R.)

**Keywords:** benign prostatic hyperplasia, prostate enucleation, transurethral resection of the prostate, prostate artery embolization, perioperative outcomes

## Abstract

**Background/Objectives:** Prostate artery embolization (PAE) has emerged as a relatively new, minimally invasive alternative for the treatment of benign prostatic hyperplasia. We aimed to compare the perioperative outcomes and trends of PAE versus transurethral resection of the prostate (TURP) and laser enucleation. **Materials and Methods:** We used the GeRmAn Nationwide inpatient Data (GRAND), provided by the Research Data Center of the Federal Bureau of Statistics, and performed multiple patient-level analyses. Patients with prostate cancer, acute hematuria, and emergent referral to the hospital were excluded. **Results:** Between 2017 and 2022, a total of 3665 PAEs were performed in Germany compared to 218,388 TURPs and 50,863 laser enucleations. Patients selected for PAE were slightly younger and presented with fewer comorbidities at baseline. The number of laser enucleations increased exponentially in these years, PAEs remained stable, whereas TURPs slightly decreased. Compared to PAE, laser enucleation was associated with higher odds of in-hospital incontinence (4.2% versus 2.7%, OR: 1.6, 95%CI: 1.3–1.9, *p* < 0.001). On the contrary, PAE was associated with lower odds of in-hospital urinary retention and shorter length of hospital stay compared to TURP (3.2% versus 7.1%, OR: 2.2, 95%CI: 1.8–2.6, *p* < 0.001, and a 2.6-day difference, 95%CI: 2.5–2.7, *p* < 0.001, respectively) and laser enucleation (3.2% versus 5%, OR: 1.5, 95%CI: 1.3–1.8, *p* < 0.001, and a 1.5-day difference, 95%CI: 1.4–1.6, *p* < 0.001, respectively). **Conclusions:** PAE offers more favorable perioperative outcomes compared to TURP and laser enucleation, but the use of this relatively new procedure has remained nearly stable in recent years.

## 1. Introduction

Prostate artery embolization (PAE) has emerged as a relatively new, minimally invasive alternative for the treatment of benign prostatic obstruction (BPO), offering a non-surgical option for patients with symptomatic BPO [1]. Previous studies have reported that PAE is less effective than TURP in improving lower urinary tract symptoms and urodynamic parameters; however, it may be associated with reduced blood loss, lower complication rates, and shorter catheterization or hospitalization time [2]. After PAE, the prostate size may be reduced by up to 50% of the initial size [3]. However, prostate volume reduction does not necessarily correlate with improvement of lower urinary tract symptoms (1). Moreover, PAE may be superior to laser enucleation in terms of safety but inferior in terms of effectiveness, also for large prostates [4].

Therefore, available guideline recommendations suggest that PAE may be offered to patients who wish to consider minimally invasive treatment options or who are at high risk of undergoing anesthesia and are willing to accept less optimal outcomes compared to the traditional BPO surgical methods [5,6]. Nevertheless, PAE continues to gain attention as an effective treatment modality for selected patients, particularly those seeking a less invasive alternative with fewer complications and quicker recovery [7]. PAE is offered mainly in selected, high-volume centers as it requires thorough training and specific infrastructures [8,9]. Moreover, different technical variations (including particle size) have been described [10]. Of note, PAE may also be offered to patients with acute or refractory to surgical treatment prostatic bleeding [11].

Despite its growing popularity, there are limited comparative data on the perioperative outcomes of PAE compared to TURP and laser enucleation [12]. In this scope, we aimed to compare the perioperative outcomes of PAE with those of TURP and laser enucleation for BPO. Through a comprehensive analysis, we also sought to provide evidence on the trends of BPO surgery.

## 2. Methods

### 2.1. GeRmAn Nationwide Inpatient Data (GRAND)

We utilized data from the German Nationwide Inpatient Data registry, provided by the Federal Bureau of Statistics in Wiesbaden, Germany. This dataset includes information on all hospitalized patients in Germany from 2005 to 2022, with the exception of military, psychiatric, and forensic cases. All presented data were accessed following the necessary approvals (LMU-4710-2022), since they are anonymized and stored at the Research Data Center of the Federal Bureau of Statistics. Our research team did not have access to individual patient data but worked with summary results provided by the Research Data Center. As per German regulations, ethical approval and patient consent were not required. These data capture only the inpatient, post-procedure hospital stay, and no information is available after the patient is discharged.

Since 2005, with the introduction of the Diagnosis Related Groups (DRG) payment system, hospitals in Germany have been required to submit patient data covering in-hospital diagnoses, perioperative outcomes, and surgical procedures to the Institute for the Hospital Remuneration System. Diagnoses and perioperative outcomes are coded using the 10th revision of the International Classification of Diseases, German Modification (ICD-10-GM), and surgical procedures are coded using the German Procedure Classification (OPS). Guidelines from the German Institute for Medical Documentation and Information ensure standardized coding procedures in Germany.

### 2.2. Data Source

For this study, we included all males with benign prostatic hyperplasia (ICD-10-GM: N40) undergoing PAE (OPS code: 8-836.kh), TURP (OPS code: 5-601.1, 5-601.0), or laser enucleation of the prostate with the holmium (OPS code: 5-601.70) or thulium laser (OPS code: 5-601.72) from 2017 until 2022. Additional diagnostic and procedural codes (ICD-10-GM and OPS) were used to gather information on coexisting conditions and inpatient complications. Patients with prostate cancer, acute hematuria, as well as those with an emergent referral to the hospital, were excluded [13].

The primary outcome of the present study was to compare the PAE with other established BPO surgical treatments in terms of perioperative morbidity (sepsis, postoperative incontinence, urinary retention, transfusion, and intensive care unit admission) and length of hospital stay. In-hospital urinary retention was defined as the need for placement of a bladder catheter after its removal during hospital stay, while urinary incontinence was defined as any involuntary urine loss after catheter removal during hospital stay. Secondary outcomes included the trends of PAE.

### 2.3. Statistical Analysis

We used multivariable logistic and linear regression models to assess the impact of different surgical treatments on in-hospital outcomes, including perioperative complications and length of hospital stay. All models were adjusted for age, diabetes, chronic renal failure, hypertension, and obesity. Categorical data were presented as frequencies with proportions, and continuous data as medians with interquartile ranges (IQR). Odds ratios (ORs) with 95% confidence intervals (CIs) were calculated, and *p*-values below 0.05 were deemed statistically significant. The statistical analyses were performed by the Research Data Center based on R scripts developed by our team (source: Research Data Center, DRG Statistics 2017–2022).

## 3. Results

### 3.1. Baseline Characteristics

Between 2017 and 2022, a total of 3665 PAEs were performed in Germany compared to 218,388 TURPs and 50,863 laser enucleations. Patients selected for PAE were slightly younger and presented with fewer comorbidities at baseline. In particular, only 9.4% of PAE patients had diabetes, compared to 20% in the TURP group and 15% in the laser enucleation group. Hypertension was common across all groups, but again least prevalent in PAE patients (39% vs. 58% TURP and 50% laser enucleation). Similarly, rates of chronic kidney disease (3.5% vs. 8.6% and 5%) and chronic obstructive pulmonary disease (2.9% vs. 6.8% and 4.3%) were markedly lower in the PAE cohort. Cardiovascular and cerebrovascular conditions, including chronic heart failure and prior stroke, were also less common among PAE patients. The baseline characteristics of the included patients are presented in Table 1.

**Figure 1:**
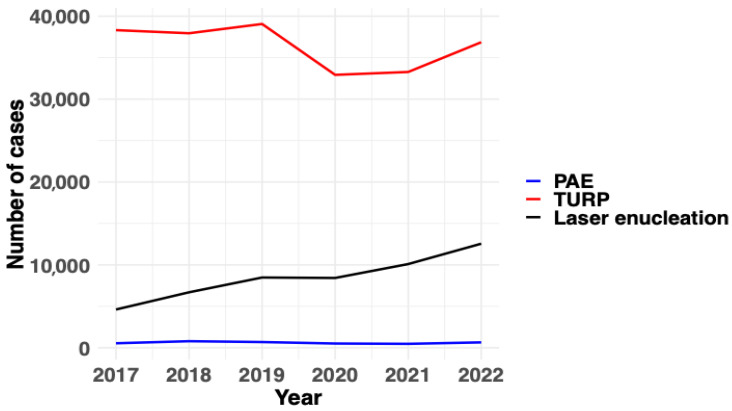
The annual trends of major surgical treatments for benign prostatic obstruction. PAE: prostate artery embolization, TURP: transurethral resection of the prostate.

**Figure 2:**
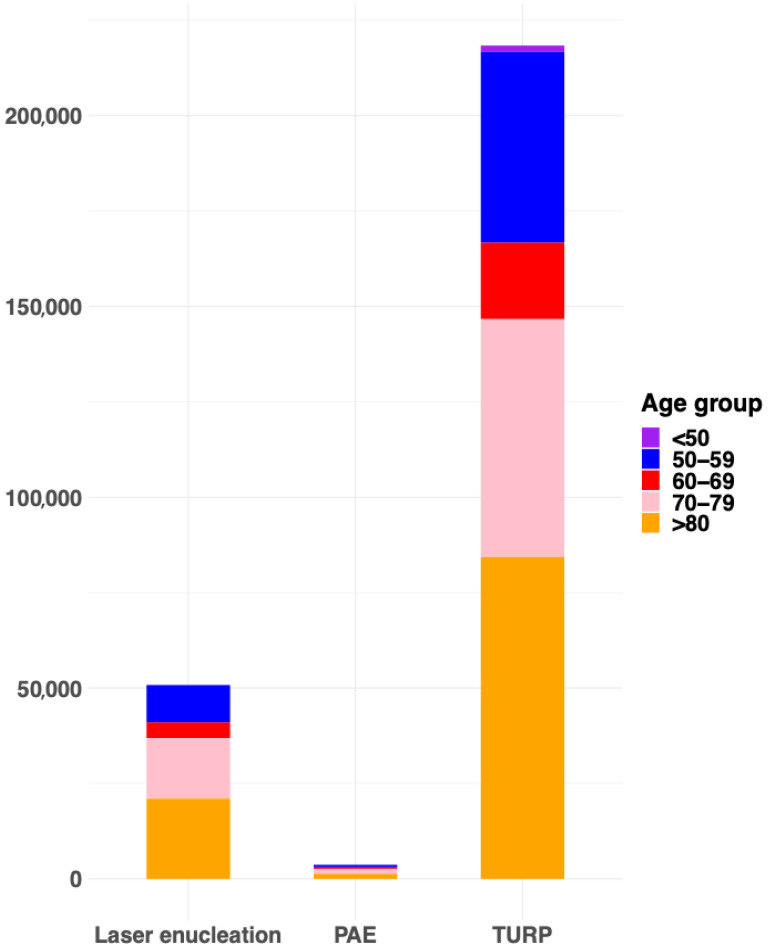
The distribution in age groups of the available surgical treatments for benign prostatic obstruction. PAE: prostate artery embolization, TURP: transurethral resection of the prostate.

In recent years, the number of laser enucleations has increased exponentially, PAE procedures have remained stable, whereas TURP has slightly decreased. The annual trends for all BPO surgical or invasive treatments are available in Figure 1. Regarding age distribution, the majority of patients across all procedures were between 70 and 79 years old. However, a higher proportion of men younger than 60 years underwent PAE compared to TURP or laser enucleation (45% versus 38% and 39%, respectively). Conversely, TURP was more frequently performed in octogenarians (23% versus 17% for PAE and 19% for laser enucleation). The age distribution of patients undergoing invasive or surgical treatments for BPO is depicted in Figure 2.

### 3.2. Perioperative Outcomes

After elective PAE, 24 (0.7%) patients were admitted to the intensive care unit, 51 (1.4%) required a transfusion, and 11 (0.3%) developed post-interventional sepsis. In the multivariate regression analysis, PAE did not differ from TURP and laser enucleation in terms of these perioperative complications. Compared to PAE, laser enucleation was associated with higher odds of in-hospital incontinence (2139 patients, 4.2% versus 98 patients, 2.7%, OR: 1.6, 95% CI: 1.3 to 1.9, *p* < 0.001). On the contrary, PAE was associated with lower odds of in-hospital urinary retention compared to TURP (119 patients, 3.2% versus 15, 600 patients, 7.1%, OR: 2.2, 95% CI: 1.8 to 2.6, *p* < 0.001), and laser enucleation (119 patients, 3.2% versus 2532 patients, 5%, OR: 1.5, 95% CI: 1.3 to 1.8, *p* < 0.001). Patients undergoing PAE were discharged earlier from the hospital compared to TURP (day difference: 2.6, 95% CI: 2.5 to 2.7, *p* < 0.001) and laser enucleation (day difference: 1.5, 95% CI: 1.4 to 1.6, *p* < 0.001). All perioperative outcomes are available in Table 2.

## 4. Discussion

The present nationwide study from Germany indicates that PAE presents favorable in-hospital perioperative outcomes compared to TURP and laser enucleation. In particular, PAE was associated with earlier hospital discharge, lower in-hospital urinary retention rates, and better in-hospital continence rates. Despite that, laser enucleation has undergone an exponential increase in Germany in the last few years, while PAE remains relatively stable. Moreover, the age distribution does not widely differ among the assessed surgical treatments for BPO in Germany.

Our findings suggest a superiority of PAE in terms of short-term postoperative outcomes, such as incontinence, urinary retention, and length of hospital stay. The observed shortening of hospital stay for PAE compared to TURP (by 2.6 days) and laser enucleation (by 1.5 days) may translate into lower healthcare costs, reduced inpatient burden, and faster recovery, which are important both for younger, active patients and for elderly patients with multiple comorbidities [14]. Similarly, the lower incidence of urinary retention in the PAE group suggests a potential benefit in postoperative recovery and catheter dependence. Indeed, prolonged postoperative catheter dependence is associated with higher infection rates and urethral strictures in the long term [15]. Nevertheless, our study focuses on perioperative, in-hospital findings rather than post-discharge morbidity, symptom resolution, or quality of life. It should be noted that previous studies indicate that PAE presents higher retreatment rates compared to TURP or laser enucleation, which could offset some of its short-term advantages [16].

The available literature suggests that PAE may be offered to patients who prefer minimally invasive treatment options over traditional BPO surgical methods or to patients who are at high risk of undergoing anesthesia [17]. However, the present epidemiological study from Germany demonstrates that patients undergoing PAE are younger and present with fewer comorbidities compared to those undergoing TURP or laser enucleation. The latter indicates that, in Germany, PAE is predominantly offered to patients who wish to consider minimally invasive treatment options and not to those who are at high risk of undergoing anesthesia [18]. Importantly, in this analysis, we excluded patients with prostate cancer, acute hematuria, and emergent hospital referrals, as their inclusion could have significantly impacted outcomes. These cases are typically associated with higher perioperative complication rates. However, their exclusion was necessary, as TURP remains the standard treatment in such scenarios [19]. In select cases of persistent hematuria, PAE may also be considered as a treatment option [20]. Conversely, laser enucleation is rarely the preferred approach in these clinical settings [21].

In our analysis, we could not provide information on the selection process for PAE over laser enucleation or TURP. Indeed, men undergoing PAE were slightly younger and carried fewer comorbidities, including lower rates of diabetes, hypertension, chronic kidney disease, and cardiopulmonary conditions. This contrasts with current guideline recommendations, which typically reserve PAE for patients at higher anesthetic risk or those seeking minimally invasive alternatives. The present findings suggest that, in Germany, PAE is predominantly chosen by patients desiring a less invasive procedure with shorter hospitalization rather than those with significant frailty or comorbidity. Conversely, TURP continues to represent the standard option across a wide spectrum of patients, particularly older individuals with more complex medical histories, while laser enucleation has increasingly gained popularity due to its safety profile and efficacy even in larger prostates and patients with comorbidities.

It should be stressed that the findings of our study are in line with previous epidemiological data suggesting a clear shift toward holmium and thulium enucleation of the prostate compared to conventional TURP due to their high safety and efficacy for patients with varying prostate sizes and comorbidities [22,23]. Although TURP remains the most common BPO surgery, it is steadily replaced by laser-based surgical treatment modalities [24,25]. Nevertheless, even though PAE seems to be a safe minimally invasive procedure, available evidence indicates that it is less effective than TURP or laser enucleation in the long term [26]. Randomized controlled trials on the matter demonstrate the superiority of TURP in terms of International Prostate Symptom Score, maximum urinary flow rate, reduction in postvoid residual urine, and reduction in prostate volume [27,28]. Accordingly, it seems that a substantial number of patients undergoing PAE require reoperation with TURP or laser enucleation in the long term. On the other hand, PAE has lower complication rates such as hematuria, urinary retention, urinary incontinence, erectile dysfunction, retrograde ejaculation, and urinary tract infection compared to TURP [29].

It should be highlighted that the cases of PAE performed in Germany in recent years were significantly lower than those of TURP and laser enucleation. Still, the sample size for PAE represents one of the largest cohorts of this procedure reported to date. The latter provides sufficient statistical power to allow meaningful comparisons of major in-hospital outcomes. Nevertheless, the relatively smaller PAE cohort may limit the robustness of direct comparisons to TURP or laser enucleation. Therefore, our findings should be interpreted as hypothesis-generating. Of note, larger prospective studies or randomized trials are mandatory to confirm our findings [30].

To our knowledge, we performed the largest study on outcomes for patients with BPO undergoing PAE compared to TURP or laser enucleation by using the administrative data of Germany. Nonetheless, our findings have certain limitations that should be noted. First, the analysis relies on retrospective billing data, which may be prone to coding errors and misclassifications. Additionally, important patient details such as laboratory results (including PSA), operative duration, prostate volume, prior conservative or surgical interventions, and urodynamic assessments were not available. Therefore, we could not adjust our multivariable regression models for further relevant factors such as lifestyle differences, medications, and other comorbidities. The study also lacks data on post-discharge morbidity, reoperations, functional outcomes, continence rates, and long-term follow-up. Thus, it leaves questions about the long-term effectiveness and potential complications of PAE compared to TURP or laser enucleation unanswered. Accordingly, given that our study is limited only to in-hospital outcomes, no safe conclusions can be drawn about the postoperative incontinence and urinary retention rates of the different surgical treatments for BPO. Importantly, the study does not provide insights into the decision-making processes or preferences of patients and surgeons in selecting surgical treatments for BPO. Based on the previous notion, it was beyond the scope of the present analysis to assess the effect of hospital- and surgeon-caseload on the perioperative outcomes. The study is also based solely on data from Germany, which limits its applicability to healthcare systems in other countries. Consequently, the generalizability of our findings is constrained, as the characteristics of patients undergoing PAE may differ from those opting for other surgical treatments for BPO.

The current real-world data suggest that PAE offers more favorable perioperative outcomes during hospital stay compared to TURP and laser enucleation. Specifically, PAE was linked to earlier hospital discharge, lower rates of urinary retention, and improved continence during hospitalization. Despite these advantages, the use of laser enucleation has seen a rapid increase in Germany over the past few years, while PAE usage has remained relatively stable due to the higher efficacy of laser enucleation in terms of long-term functional outcomes. Still, all BPO surgical procedures were associated with low perioperative complications during hospital stay. Nevertheless, it should be highlighted that the findings of the present study should be interpreted with caution due to its important limitations. Overall, each BPO surgical procedure comes with its own advantages, disadvantages, and specific indications, all of which must be carefully evaluated and discussed with patients. Understanding the trends and perioperative outcomes of BPO surgery is of utmost importance to enhance clinical decision-making and patient outcomes.

## Figures and Tables

**Table 1:** Baseline characteristics of the included patients. Variables are presented as median (interquartile range) or frequencies with proportions. The bold cells indicate statistically significant values. COPD: chronic obstructive pulmonary disease, TURP: transurethral resection of the prostate.

Characteristic	Overall, n = 272,916	Prostate Artery Embolization, n = 3665	TURP, n = 218,388	Laser Enucleation, n = 50,863	*p*-Value
Age (years)	71 (65–78)	69 (63–76)	72 (65–78)	71 (65–77)	**<0.001**
Diabetes	52,277 (19%)	345 (9.4%)	44,107 (20%)	7825 (15%)	**<0.001**
Chronic heart failure	10,280 (3.8%)	68 (1.9%)	8963 (4.1%)	1249 (2.5%)	**<0.001**
COPD	17,108 (6.3%)	105 (2.9%)	14,792 (6.8%)	2211 (4.3%)	**<0.001**
Chronic kidney disease	21,514 (7.9%)	130 (3.5%)	18,861 (8.6%)	2523 (5%)	**<0.001**
Cerebrovascular disease	4521 (1.7%)	30 (0.8%)	3968 (1.8%)	523 (1%)	**<0.001**
Dementia	3648 (1.3%)	16 (0.4%)	3226 (1.5%)	406 (0.8%)	**<0.001**
Hypertension	153,076 (56%)	1445 (39%)	126,224 (58%)	25,407 (50%)	**<0.001**
Obesity	14,417 (5.3%)	147 (4%)	12,424 (5.7%)	1846 (3.6%)	**<0.001**
Year of surgery					**<0.001**
2017	43,480 (16%)	543 (15%)	38,320 (18%)	4617 (9.1%)	
2018	45,430 (17%)	794 (22%)	37,944 (17%)	6692 (13%)	
2019	48,231 (18%)	690 (19%)	39,064 (18%)	8477 (17%)	
2020	41,860 (15%)	513 (14%)	32,926 (15%)	8421 (17%)	
2021	43,847 (16%)	474 (13%)	33,273 (15%)	10,100 (20%)	
2022	50,068 (18%)	651 (18%)	36,861 (17%)	12,556 (25%)	
Age group					**<0.001**
<50	1819 (0.7%)	21 (0.6%)	1682 (0.8%)	116 (0.2%)	
50–59	24,606 (9.0%)	407 (11%)	20,090 (9.2%)	4109 (8.1%)	
60–69	79,485 (29%)	1258 (34%)	62,310 (29%)	15,917 (31%)	
70–79	106,699 (39%)	1351 (37%)	84,359 (39%)	20,989 (41%)	
>80	60,307 (22%)	628 (17%)	49,947 (23%)	9732 (19%)	

**Table 2:** Multivariable logistic regression analysis based on the surgical option for treatment of benign prostatic obstruction. All models are adjusted for age, diabetes, chronic renal failure, hypertension, and obesity. The bold cells indicate statistically significant *p*-values. CI: confidence interval, ICU: intensive care unit, TURP: transurethral resection of the prostate.

Outcome	Prostate Artery Embolization		TURP		Laser Enucleation	
	Cases	Estimate (95% CI), *p*-Value	Cases	Estimate (95% CI), *p*-Value	Cases	Estimate (95% CI), *p*-Value
ICU admission	24 (0.7%)	—	1668 (0.8%)	0.97 (0.66, 1.5), 0.9	392 (0.8%)	1.1 (0.73, 1.7), 0.7
Transfusion	51 (1.4%)	—	3932 (1.8%)	1.1 (0.8, 1.4), 0.7	678 (1.3%)	0.9 (0.67, 1.2), 0.4
Sepsis	11 (0.3%)	—	1238 (0.6%)	1.6 (0.95, 3.2), 0.1	171 (0.3%)	1.1 (0.6, 2.1), 0.9
Postoperative incontinence	98 (2.7%)	—	6698 (3.1%)	1.1 (0.88, 1.3), 0.5	2139 (4.2%)	**1.6 (1.3, 1.9), <0.001**
Urinary retention	119 (3.2%)	—	15,600 (7.1%)	**2.2 (1.8, 2.6), <0.001**	2532 (5%)	**1.5 (1.3, 1.8), <0.001**
Length of hospital stay	2 (1–2)	—	5 (3–6)	**2.6 (2.5, 2.7), <0.001**	4 (3–5)	**1.5 (1.4, 1.6), <0.001**

## Data Availability

The original contributions presented in the study are included in the article; further inquiries can be directed to the corresponding authors.
